# Genome Sequence of a Novel Virus of the Species Human Adenovirus D Associated with Acute Gastroenteritis

**DOI:** 10.1128/genomeA.00068-12

**Published:** 2013-01-15

**Authors:** Yuki Matsushima, Hideaki Shimizu, Atsuko Kano, Etsuko Nakajima, Yoko Ishimaru, Shuvra Kanti Dey, Yuki Watanabe, Fuyuka Adachi, Kohnosuke Mitani, Tsuguto Fujimoto, Tung Gia Phan, Hiroshi Ushijima

**Affiliations:** a Division of Virology, Kawasaki City Institute of Public Health, Oshima, Kawasaki-ku, Kawasaki-shi, Kanagawa, Japan; b Division of Microbiology, Department of Pathology and Microbiology, Nihon University School of Medicine, Oyaguchi Kamicho, Itabashi-ku, Tokyo, Japan; c Division of Gene Therapy, Research Center for Genomic Medicine, Saitama Medical School, Yamane, Hidaka-shi, Saitama, Japan; d Infectious Disease Surveillance Center, National Institute of Infectious Diseases, Toyama, Shinjuku-ku, Tokyo, Japan; e Blood Systems Research Institute, San Francisco, California, USA

## Abstract

A novel virus of the species human adenovirus D, HAdV-67 (P-New/H9/F25), was first isolated from diarrheal feces of six children in Dhaka City, Bangladesh. The genome of this novel virus may be composed of multiple recombinations among HAdV-9, HAdV-25, HAdV-26, HAdV-33, HAdV-46, and an unknown human adenovirus D which was an origin of HAdV-67.

## GENOME ANNOUNCEMENT

Human adenoviruses (HAdVs) infect humans of all age groups and are significant pathogens that cause a wide range of clinical diseases, including epidemic keratoconjunctivitis, acute respiratory illnesses, and acute gastroenteritis (1, 2, 3). Sixty-five types, including candidates from HAdV-55 to HAdV-65, have been identified and classified into seven species, A to G, each of which shows different organotropisms.

A novel virus of the species human adenovirus D was identified from diarrheal feces of six children in Bangladesh. This novel virus has been approved as a new type of HAdV-67 by the Human Adenovirus Working Group. For amplifying the genome fragments of HAdV-67, the previously published sequence of HAdV-9 was used to design primers. A complete genome of HAdV-67 was sequenced using primer walking. For the terminal genome sequence, the 5′ terminus of full-length DNA was phosphorylated with T4 polynucleotide kinase (Takara, Shiga, Japan). Afterward, a blunt-end EcoRI-NotI-BamHI adaptor (Takara, Japan) was ligated using the DNA Ligation Mighty Mix kit (Takara, Japan).

A complete genome of HAdV-67 was 35,075 bp in length and had an overall base composition of 22.47% (A), 20.48% (T), 28.56% (G), and 28.49% (C). Like those of other HAdV-Ds, the genome of HAdV-67 was predicted to encode 38 proteins in four early, two intermediate, and five late transcriptional regions. The inverted terminal repeat (ITR) of HAdV-67 was 123 bp in length and contained a conserved CATCATCAAT motif. Additionally, its transcription factor DNA binding sites, such as those for NFIII/OctI (ATGCAAAT) at nucleotide positions 42 to 49, SP1 (AGGGCGG) at nucleotide positions 64 to 70, and ATFs (TGACGT) at nucleotide positions 107 to 112, were present within the ITR. No nuclear factor I (NFI) binding sites, which exist in some HAdV-Ds (HAdV-9, HAdV-15, HAdV-19, HAdV-26, HAdV-29, HAdV-37, HAdV-53, HAdV-56, and HAdV-58), were identified in HAdV-67. In a phylogenetic analysis, HAdV-67 clustered with HAdV-9 (loops 1 and 2 of the hexon gene), HAdV-46 (HVL1 of the penton base gene), HAdV-33 (genes for a DNA binding protein and 100,000-molecular-weight protein [100K protein] in the L4 region), HAdV-26 (genes for CR1-α, gp19K, CR1-β, and CR1-γ in the E3 region), and HAdV-25 (fiber gene). Interestingly, HAdV-67 showed low similarities to other HAdV-Ds in the RGD loop of the penton base gene. These results suggested that the novel genome might be composed of multiple recombinations among HAdV-9, HAdV-25, HAdV-26, HAdV-33, HAdV-46, and an unknown human adenovirus D which was an origin of HAdV-67. The species HAdV-A and -F are primarily associated with acute gastroenteritis, whereas HAdV-Ds cause acute gastroenteritis in AIDS patients (4, 5, 6, 7, 8). We previously reported a novel HAdV-D (HAdV-65) isolated from diarrheal feces of infants without immunodeficiency (9). In this study, a novel HAdV-D, HAdV-67 (P-New/H9/F25), was found in acute gastroenteritic children who were previously healthy, indicating its potential threat associated with this illness. Moreover, this study demonstrates the importance of identifying recombinant regions within the genomes of HAdVs.

### Nucleotide sequence accession number.

The GenBank accession number for HAdV-67 (P-New/H9/F25) is AP012302.
